# Developing and validating an instrument to assess non-hospital health centers’ preparedness to provide initial emergency care: a sequential exploratory mixed-method study

**Published:** 2019-08

**Authors:** Mehrdad Amir Behghadami, Homayoun Sadeghi-Bazargani, Masoumeh Gholizadeh, Farzad Rahmani

**Affiliations:** ^*a*^MSc in Health Services Management, Iranian Center of Excellence in Health Management (IceHM), Department of Health Service Management, School of Health Services Management and Medical Informatics, Tabriz University of Medical Sciences, Tabriz, Iran.; ^*b*^Department of Statistics and Epidemiology, Faculty of Health, Tabriz University of Medical Sciences, Tabriz, Iran.; ^*c*^Iranian Center of Excellence in Health Management (IceHM), Department of Health Service Management, School of Health Services Management and Medical Informatics, Tabriz University of Medical Sciences, Tabriz, Iran.; ^*d*^Emergency Medicine Department, Sina Medical Research and Training Hospital, Tabriz University of Medical Sciences, Tabriz, Iran.

## Abstract

**Background:**

Basic emergency management in urban and rural areas is a critical challenge. The main aim of the study was to develop, validate, and pilot an instrument which can be used to measure the preparedness of non-hospital health centers to provide initial emergency care. This study was designed based on a sequential exploratory mixed-method in two qualitative and quantitative phases. In the qualitative phase, the literature systematic review and qualitative.

**Methods:**

(Focus Group Discussions (FGDs) and Semi-Structured Interviews (SSIs) with the target group) were applied to identify the domains and items. Purposive sampling method was used to identify participants in the SSIs and FGDs. Sampling was done until theoretical saturation. Interview data were analyzed using framework analysis and then, the results of both literature systematic review and qualitative studies were triangulated by a panel of experts to define the domains and items to be included in the instrument. In the quantitative phase, Content Validity Ratio (CVR) and Content Validity Index (CVI) of the instrument were performed. The CVR was assessed based on the necessary criterion. The CVI was assessed using modified Kappa coefficient (modified CVI) based on clarity and relevance criteria.

**Results:**

In the qualitative phase, primary version of the instrument containing 155 items related to assessing preparedness of non-hospital health centers was generated. In the quantitative phase, item reduction was applied and the final version of the instrument containing 120 items was developed. These items were classified in 10 domains which include: fundamental infrastructures, medical supplies and equipment, human resources, emergency medicines, clinical interventions, maintenance of equipment, medicine storage capability, educating and training, infection control, and quality control. The CVI and kappa modified were calculated 0.80 and 0.80 for relevance and .91 and 0.91 for clarity, respectively.

**Conclusions:**

This study provided a standard and valid instrument which can be used to assess preparedness rate of non-hospital health centers to deliver initial emergency care.

**Keywords:**

Non-hospital health centers, Preparedness, Assess, Emergency care, Validity, Sequential exploratory mixed-method study

